# Asymmetric cell division of mammary stem cells

**DOI:** 10.1186/s13008-021-00073-w

**Published:** 2021-09-29

**Authors:** Shaan N. Chhabra, Brian W. Booth

**Affiliations:** 1 Wofford College, Spartanburg, SC 29303 USA; 2 Department of Bioengineering, Head-Cellular Engineering Laboratory, 401-1 Rhodes Engineering Research Center, Clemson University, Clemson, SC 29634 USA

**Keywords:** Asymmetric cell division, Cancer stem cells, Mammary stem cells, Molecular mechanisms

## Abstract

Somatic stem cells are distinguished by their capacity to regenerate themselves and also to produce daughter cells that will differentiate. Self-renewal is achieved through the process of asymmetric cell division which helps to sustain tissue morphogenesis as well as maintain homeostasis. Asymmetric cell division results in the development of two daughter cells with different fates after a single mitosis. Only one daughter cell maintains “stemness” while the other differentiates and achieves a non-stem cell fate. Stem cells also have the capacity to undergo symmetric division of cells that results in the development of two daughter cells which are identical. Symmetric division results in the expansion of the stem cell population. Imbalances and deregulations in these processes can result in diseases such as cancer. Adult mammary stem cells (MaSCs) are a group of cells that play a critical role in the expansion of the mammary gland during puberty and any subsequent pregnancies. Furthermore, given the relatively long lifespans and their capability to undergo self-renewal, adult stem cells have been suggested as ideal candidates for transformation events that lead to the development of cancer. With the possibility that MaSCs can act as the source cells for distinct breast cancer types; understanding their regulation is an important field of research. In this review, we discuss asymmetric cell division in breast/mammary stem cells and implications on further research. We focus on the background history of asymmetric cell division, asymmetric cell division monitoring techniques, identified molecular mechanisms of asymmetric stem cell division, and the role asymmetric cell division may play in breast cancer.

## Introduction

Significant features of somatic stem cells are their capability to self-renew and to give rise to progeny that will undergo differentiation. Asymmetric cell division (ACD) produces two daughter cells with differing cellular fates; one stem cell and one differentiating cell. Alternatively, stem cells can undergo proliferating symmetric cell divisions which give rise to two daughter stem cells. A balance between these two forms of division is necessary for normal development and homeostasis.

This comprehensive literature review of ACD, including mechanistic results, was formulated from extensive Pubmed and Google web searches. Multiple search terms were included in many of the bibliographic searches. The years included span from 1959 to 2021.

### Immortal strand theory

**Fig. 1:**
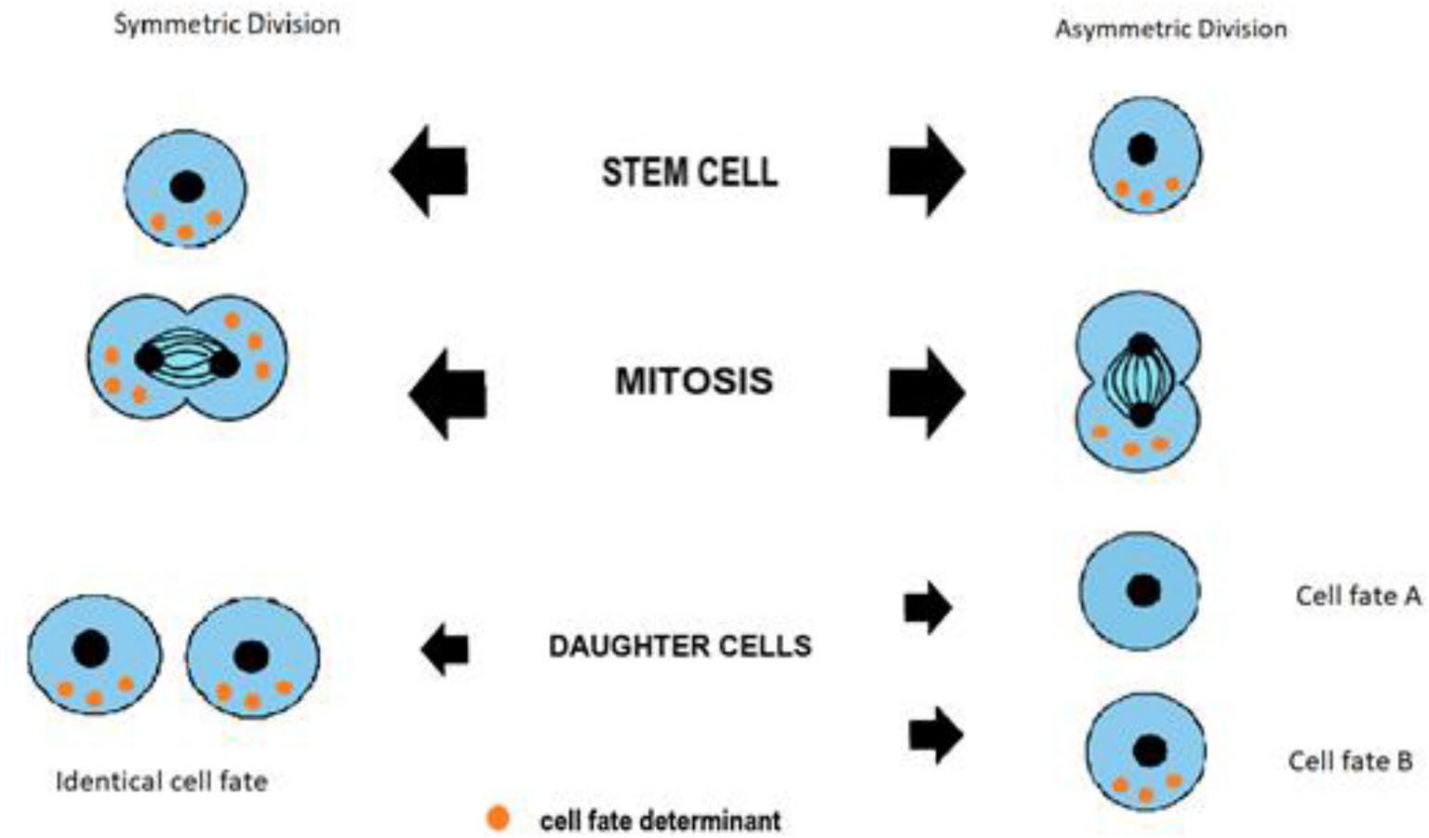
Asymmetric and symmetric division of stem cells. Chromosomes and cell fate determinants have different fates depending on cell division mechanism

Cairns and Potten first noted in 1978 that intestinal epithelial cells have failed to produce carcinomas at a rate commensurate with the number of divisions they undergo throughout their lives [1]. This discovery contributed to the formulation of the immortal DNA strand hypothesis that suggests that somatic stem cells asymmetrically segregate their DNA thereby retaining an “immortal” DNA template while transferring newly developed chromatids to daughter cells (Fig. 1). Somatic stem cells divide rarely and maintain a state of relative quiescence. Subtypes of adult mammalian stem cells can be held in this primed, quiescent state and subsequently reactivated to restore homeostasis after tissue injury [2]. According to the ‘immortal DNA strand’ model, adult stem cells retain template DNA strands in each asymmetric division to prevent mutations from accumulating during the DNA replication process [3]. The stem cells of somatic tissues are thought to protect themselves from mutation and malignancy by selectively segregating their template DNA strands.

**Fig. 2:**
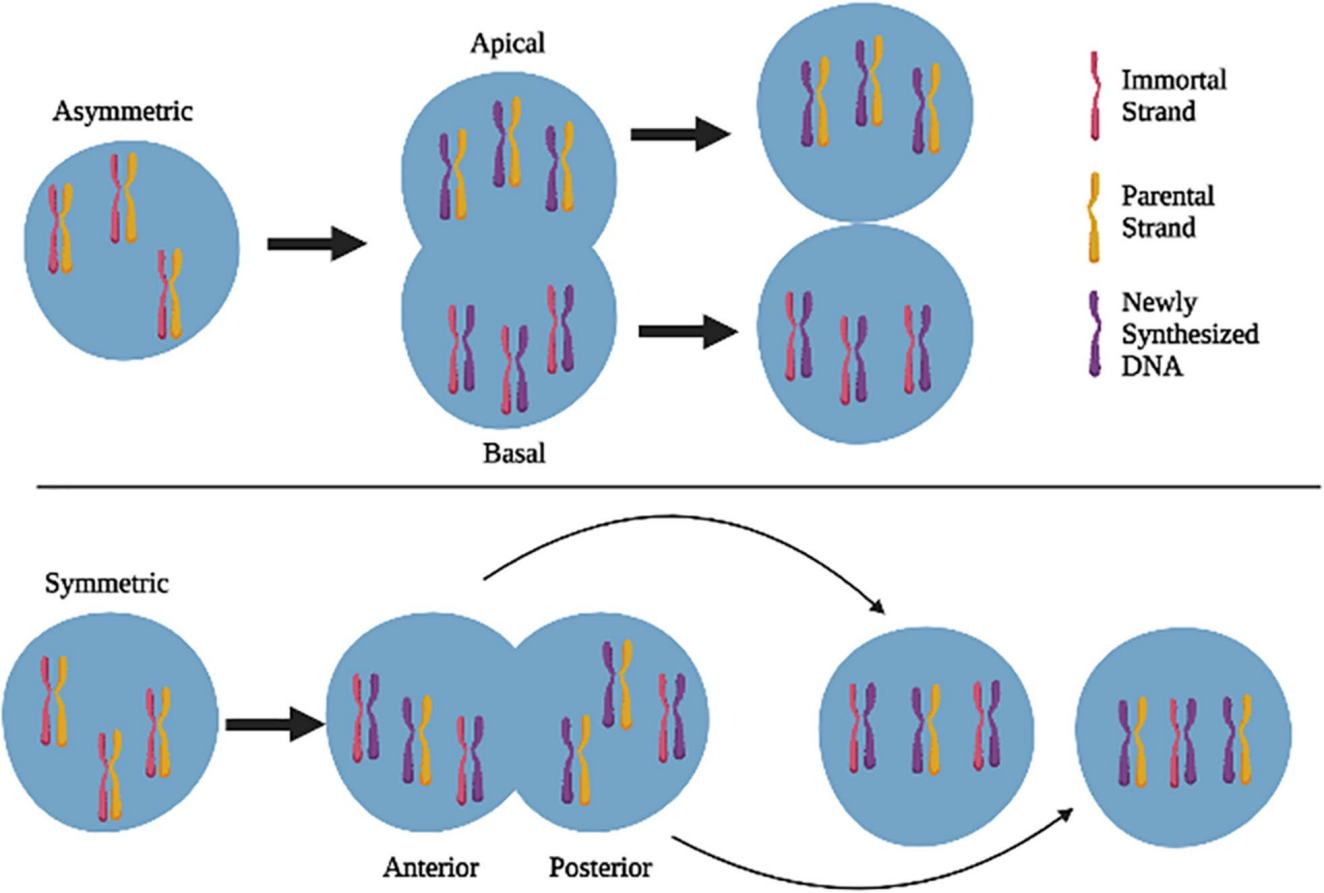
DNA segregation during asymmetric division and symmetric division. During asymmetric division the stem cell divides vertically, the new stem cell is located basally in relation to the new daughter cell that is in the apical position. The immortal DNA strands and associated newly synthesized DNA segregate to the basal stem cell while the new daughter cell contains newly synthesized DNA and the parental DNA. During symmetric cell divisions the two new stem cells each contain randomly distributed DNA containing both immortal strands and parental strands

In order to test the immortal strand theory stem cells were labeled with thymidine analogs during symmetric divisions in classic pulse-chase experiments. Mammary stem cells divide symmetrically during pubertal mammary gland growth and development. The labeled stem cells maintained the nuclear labels once they initiated asymmetric divisions resulting in label retaining cells (LRCs) (Fig. 2). The first demonstration of LRCs in the mouse mammary gland was in 1996 by Zeps [4] and this was confirmed in 2005 by Smith [5]. Both published evidence that a subset of mouse mammary epithelial cells could retain and release DNA label in a manner consistent with the immortal DNA strand mechanism. [^3^H]-thymidine (^3^HTdR) was used to mark self-renewing mammary epithelial stem cells that were formed during allometric development of the mammary ducts in pubertal females. [^3^H]TdR-label retaining epithelial cells (LREC) were located amid the epithelium of growing glands after a lengthy search during which much of the branching duct morphogenesis was completed. The use of a different marker, 5-bromodeoxyuridine (5BrdU), to label newly produced DNA in these glands resulted in the emergence of doubly marked nuclei in a substantial percentage of the LREC. Populations of LRECs also express estrogen receptor (ER) or progesterone receptor (PR) indicating that all LRECs are not stem cells but a subpopulation of LRECs are cells that exited the cell cycle following incorporation of the nuclear label and differentiated [6]. Label-retaining cells in the mammary stroma, on the other hand, did not incorporate 5BrdU during the pulse, indicating that they were not currently undergoing a cell cycle [7].

Mammary stem cells also divide symmetrically during the epithelial expansion that occurs during pregnancy. LRECs that were labeled during puberty persist through pregnancy and involution suggesting a long-lived stem cell population in the mammary gland [7]. Additionally, new LRECs were labeled during the symmetric stem cell divisions that occur during pregnancy and these newly labeled LRECs also persist through mammary gland remodeling that occurs during involution. These findings show that during asymmetric divisions, mammary LREC retain their [^3^H]TdR-labeled template DNA strands while passing newly generated 5BrdU-labeled DNA to their progeny [5].

During ACD, cells must establish asymmetry/polarity (Fig. 1). Varying degrees of intrinsic versus extrinsic cues guide these cells during the establishment of polarity. Stem cells use intracellular machineries to divide in a desired orientation in the context of the asymmetry/polarity. Recent studies have extended our awareness of asymmetric cell division mechanisms, exposing the previously known complexity in the formation of cellular and/or environmental asymmetry, which ensures binary outcomes of the fate determination [4]. Much of what we know about ACD regulation comes from studies of neuroblasts (NBs), the stem-like cells in the central nervous system (CNS) of *Drosophila melanogaster*. Studies on *Drosophila* NBs and male germline stem cells were performed in the early 1990s, which ultimately laid the foundation for a better understanding of basic ACD principles. NBs divide asymmetrically to self-renew. NB ACD generates a secondary precursor cell called a mother ganglion cell which in turn divides into neurons and glia [8]. Even in isolated cultures, NBs have the ability to undergo ACD making them the paradigm of intrinsic ACD. Studies of NBs have characterized cortex polarization, positioning of the mitotic spindle across the polarity axis, and the unequal partitioning of the cellular components. These characteristics determine the character of intrinsic ACD.

## Molecular mechanisms of asymmetric stem cell division

In multicellular organisms, the maintenance of tissue homeostasis specific control of somatic stem cell activity is essential [4]. In order to ensure replacement of damaged cells the expansion rate of stem and progenitor cells must at any time be closely related to tissue demands [9]. Maintaining a balance of stem and non-stem cells ensures long-term tissue homeostasis. Failure to maintain homeostasis leads to pathogenesis such as tumorigenesis and/or tissue degradation [4, 10]. Since one stem cell and one differentiating cell is the result of an asymmetric self-renewal division, this cycle helps to preserve stem cell volume [11]. ACD can be seen as advancing in four major stages. First, a polarity axis must be specified. Second, determinants of the cell fate must be located by specified cell poles. Next, the spindle apparatus is guided to one of the two daughter cells according to the pre-established axis to ensure the differentiation of the cell-fate determinants during mitosis. Last, successful separation of the two daughter cells by cytokinesis [10].

### Cell polarity/asymmetry

ACD of stem cells relies on asymmetric aesthetics of cells (cell polarity) inside the cell and the local cell environment [11]. Examples of mechanisms used to specify cell polarity and explicit asymmetric divisions are asymmetric specificity of cell–cell junctions, intrinsic determinants of cellular fate, and location of the stem cell within a particular microenvironment (niche) [12]. Cell division is controlled by intrinsic or extrinsic mechanisms. Cells like *Drosophila* NBs possess an underlying axis of polarity during the intrinsic mode of asymmetric division which allows for asymmetrical localization of cell fate determinants and other proteins within the cells further allowing the mitotic spindle to orient itself along the same axis of polarity. This results in only one daughter cell inheriting the aforementioned determinants when the cell divides resulting in different fates [11]. On the other hand, during the extrinsic mode of asymmetric division, cellular precursors receive external signals to self-renew allowing the mitotic spindle to be oriented perpendicularly to those signals ensuring that only one of the daughter cells continues to receive these signals resulting in different fates of those daughter cells [13].

### Alignment of the mitotic spindle

In both symmetrically and asymmetrically dividing cells, spindle orientation is an essential process [14]. Spindle orientation determines morphology of the tissue (ex., lung branching and epidermal stacking) and cellular differentiation (symmetric to asymmetric) [15–17]. Proper alignment and orientation of the mitotic spindle with regard to the cell polarity axis must be accomplished during asymmetric stem cell division to ensure that cell fate determinants are properly divided into only one daughter cell. The mitotic spindle aligns parallel to the polarity axis in asymmetrically dividing cells in a way that the determinants of basal cell fate are separated into only one of the daughter cells. This guarantees different fates for both the cells. On the other hand, many epithelial cells differentiate symmetrically and orientate their mitotic spindles perpendicular to the primary apical-basal polarity axis resulting in both daughter cells remaining within its epithelium. Symmetric cell division is also used by stem cells to expand their population. It is considered essential for preserving epithelial integrity, since misoriented divisions can produce daughter cells that reside either above or below the epithelial layer. Such extra-epithelial cells are isolated from their environment and may promote the development of tumors [18]. Proper development and maintenance of many epithelial tissues across a variety of organisms involves robust regulation of mitotic spindle positioning [19].

### Fate determinants and their role in ACD

Proteins, such as transcription factors, play an important role in cellular fate. These proteins are inherited differentially by the daughter cells to generate a distinction in cell fate (Fig. 2) [20]. Protein determinants are separated asymmetrically by the activity of certain adaptor proteins leading up to cell division. They include determinant proteins such as Numb, Prospero, and Brat. Determinant proteins can reside in the basal plasma membrane [21]. Numb is the first recognized cell fate determinant that partitions differentially between two daughter cells to drive their distinct developmental identities [22]. Numb's role as a determinant protein was associated with the ability to bind and biologically antagonize Notch, a membrane receptor that also determines cell fate [23]. These determinants are accompanied by a mitotic spindle alignment that assures that cell division will lead to asymmetric division of protein determinants between the two daughter cells [24, 25]. One daughter's cell is self-renewed to continue as a stem cell, and the other daughter cell differentiates [26].

**Fig. 3:**
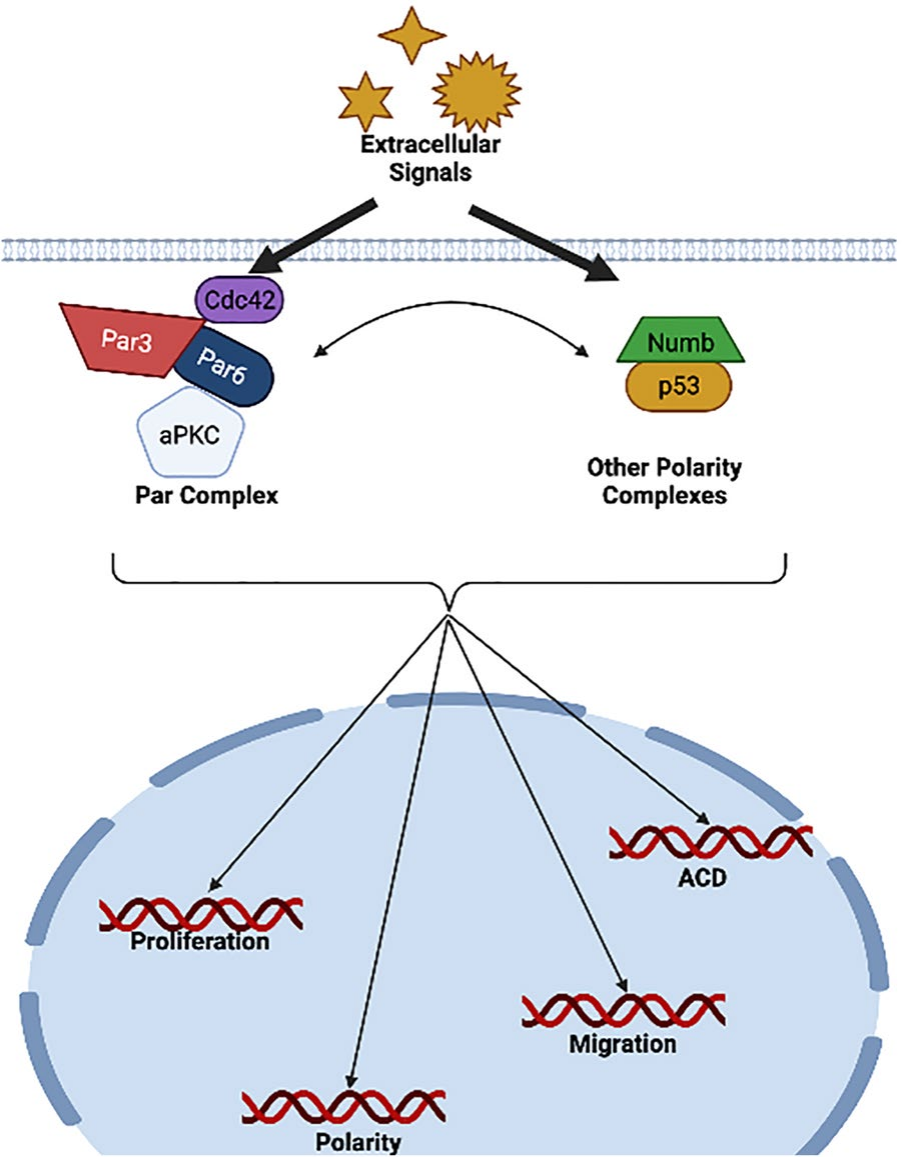
Multiple polarization processes that are part of normal cell physiology are influenced by Par complex activity and additional cell polarity complexes including Numb and p53. To ensure normal cell homeostasis, the complex integrates extracellular stimuli into the polarized cell program and interprets spatial signals resulting in gene expression

ACD relies on the partitioning of the Par3/Par6/aPKC polarity proteins and other fate determinants that are capable of conferring a given fate on the cell inheriting the proteins [27]. Par3 (Bazooka, Baz in Drosophila), Par6, and atypical protein kinase C (aPKC) are multi-domain proteins capable of interacting with each other as well as interacting with a variety of other cell polarity-regulating proteins [28] The PAR protein network, which polarizes a wide range of animal cell types, is made up of the conserved polarity effector proteins PAR-3, PAR-6, CDC-42, and aPKC. The basic principles of the Par complex assembly and its activities in cell polarity in several cell types have been established in recent decades [29–32] (Fig. 3). However, it is unclear how the Par proteins are recruited and highly concentrated in relatively confined membrane regions to establish polarity.

Neuroblasts' exceptional capability to undergo ACDs, even in isolated cultures, has made them the model for intrinsic ACDs. Intrinsic ACDs are defined by mitotic cells' ability to cell autonomously which includes the polarity proteins Par3/Par6/aPKC, as well as fate determinants, or molecules capable of conferring a specific fate on the cell that inherits them. The transcription factor Prospero, the endocytic Notch inhibitor Numb, and the tumor suppressor Brat [33, 34] have all been shown to have this action in neuroblasts. Their activities include polarizing the cortex, aligning the mitotic spindle along the polarity axis, and unequally partitioning cellular components.

## Stem cells of the mammary gland

Adult mammary stem cells (MaSCs) are a diverse group of cells that are responsible for the growth and development of the gland during puberty and expansion during any subsequent pregnancy. Understanding regulation of MaSCs is an important area of research, with a possibility that MaSCs may be susceptible targets for transformation to tumor cells. Stem cells inside the mammary gland have been proposed to be the source of several forms of breast cancer [35]. Here we summarize the identity of MaSCs, their regulation, and the documentation of their function as breast cancer sources.

### Asymmetric cell division in mammary gland

Over the last few years significant advances have been made which have laid the foundation for understanding how these MaSCs are regulated in both humans and mice during developmentally critical processes. Since MaSCs are candidates for breast cancer transformation, learning how MaSCs are controlled is a very significant research field [35].

The mammary gland consists of epithelial cells, adipocytes, as well as other stromal and nerve cells that function together during nursing to achieve the primary objective of milk production. The epithelial component is divided into alveolar luminal cells that produce milk during lactation, and luminal ductal cells that form the inner ducts allowing secreted milk to flow to the nipple. The ductal luminal cells face the lumen and are surrounded by an outer layer of myoepithelial cells which contract to help excrete milk [35].

The mammary gland ductal system resembles a tree made up of hollow branches. Increased ovarian output of estrogen and increased pituitary gland growth hormone synthesis at the initiation of puberty (among several other systemic factors) facilitates cell division and the formation of terminal end bud (TEBs) in rodents. TEBs, the bulb-shaped formations at the ends of elongating lactiferous ducts, are highly proliferative structures that are involved in development of the mammary glands [36]. The TEB structure is unique to the mammary gland and consists of two main compartments, the cap and body cell layers, which guide the growth of the ducts through the fat pad. The least differentiated cells are the cap cells at the front of the TEBs. Cells become more distinguished in the narrowing area (myoepithelial progenitors) and the terminal end bud neck area [36]. Cap cells that lead the tip of the TEB are a source for regenerative mammary stem cells as they have a greater capacity to shape a whole ductal tree when transplanted as a diluted population [37]. The cap cell layer contains MaSCs which are able to undergo symmetric and asymmetric self-renewal [38–40]. Cap cells' asymmetric mitotic divisions take place perpendicularly to the basement membrane as one of the daughter cells abandons the contacts with the original niche (dividing cells at the bottom of the bud). The symmetrical mitotic divisions of cap cells take place parallel to the basement membrane and guarantee the preservation of the identity of stem cells and their expansion in numbers. The juvenile mammary gland at the onset of puberty undergoes growth and differentiation at the end of the terminal buds. A legacy of either myoepithelia or luminal epithelia can be taken up by the cap cell layer covering the TEB. The TEBs, however, are considered to be only a temporary niche, since TEBs are transient structures that disappear once the duct reaches the end of the fat pad [41].

The presence of a resident stem cell population has long been recognized because of the incredible regenerative potential of the mammary gland [42]. DeOme et al*.* successfully demonstrated the presence of mammary stem cells with transplantation assays where they showed that epithelial segments taken from the mature gland were able to regenerate an entire functioning gland when transplanted onto a cleared fat pad of juvenile recipients regardless of where they originated in the donor gland, parity status, or age of the donor [43].

Alveolar cells generate milk proteins during pregnancy and lactation [44]. The capability to proliferate and expand the mammary gland through the cycles of pregnancy, lactation, and tissue remodeling associated with glandular involution during the entire lifetime of a woman has been attributed to MaSCs [45]. The roles performed by these cells include (1) giving rise to adult mammary gland tissues during development; (2) enabling tissue expansion and mammary gland remodeling during periods of breastfeeding, milk production, and involution; and (3) serving as a repair aid in the event of tissue injury.

It is critical to maintain an apical-basal epithelial polarity for the functioning of the organ [46]. Disruption of the polarized architecture is one of the hallmarks of breast cancer development [47]. Establishing an apical-basal polarization in mammary cells demands that Par proteins be recruited on the apical side and the Scribble complex on the basolateral side [48]. Par3, a scaffolding protein, is dedicated to the spatial localization and recruitment of several signaling effectors to the apical side. Scribble, a scaffolding protein, was originally identified in *Drosophila melanogaster* as a DLGAP5 (Discs Large) and also LLGL1 (Lethal Giant Larvae) tumor suppressor [49]. In humans, Scribble serves as a membrane protein involved in migration, polarity, and reproduction of epithelial cells [49, 50].

The nature of the cells that serve as targets of transformation has been a source of debate [51]. Stem cells and progenitor cells are regarded to be the cells-of-origin of many cancers. The defining features of replicative potential and their long cellular lifespan contribute to their susceptibility for accumulating mutations [52]. The tumor suppressor gene, BRCA1, is often mutated in the germ line, increasing the risk of basal-type breast cancer significantly. Previous research had suggested that BRCA1 is a key regulator of mammary stem cell fate, and that women with germ-line BRCA1 mutations exhibited an increase in BRCA1 mutant mammary stem/progenitor cells in their breast tissues [53]. As a result, it was assumed that BRCA1-mutant breast cancer arose from a basal progenitor/stem cell. However, a new flurry of publications calls this concept into question. Molyneux and colleagues developed a new method to determine the cell-of-origin for BRCA1-mutant breast cancer using a conditional mouse model of BRCA1 deficiency in which *Cre* recombinase-dependent deletion of exons encoding the BRCA1 protein's C-terminus, coupled with p53 heterozygosity, resulted in tumor development. Cell surface antigen profiles were utilized to differentiate three distinct mammary epithelial populations. They found that *Blg* (beta lactoglobulin) activity is highest in a CD24 +/High Sca-1 ER cell population, indicating that a luminal ER progenitor cell is the cell of origin for BRCA1-mutant basal-like tumors. This convincing data demonstrated that luminal progenitors may serve as the cellular origins of both luminal- and basal-like human breast cancer, and that the different genetic mutations that occur during luminal progenitors' transition are likely determinants of the tumor phenotypes [54]. It's likely that luminal progenitor cells with the BRCA1 mutation experience some dedifferentiation, allowing the return to a bipotent or possibly oligopotent stem–cell state. Luminal progenitor cells would be able to produce carcinomas with a basal phenotype in this case (through conversion to a bipotent progenitor or stem cell) [54, 55]. It has also been demonstrated that regardless of BRCA1, luminal progenitors can give rise to basal-like breast tumors in response to oncogenic insults [56–58].

#### Cell signaling

Cells must be able to receive and process information that originates outside of the cell to enable the cells to respond and adapt to their environment. Intercellular signaling regulates essential cellular behavior by initiating diverse responses between cells, and between cells and the extracellular matrix. Intracellular signaling pathways regulate coordination and communication between the cell surface and nucleus [59].

Mouse MaSC markers—CD24 (heat-stable antigen), CD29 (β1-integrin), and CD49f (α6-integrin) [60] that are used to purify MaSC populations were first reported in 2006 [60, 61]. Based on the purification approaches for MaSCs, subsequent experiments were undertaken to unravel the molecular mechanisms governing MaSC stemness and differentiation along a specific lineage. Next, we review the role of these signaling pathways in the normal development of the mammary gland and the evidence that deregulation of these pathways is important in mammary carcinogenesis.

### Hedgehog

Hedgehog (Hh) signaling is essential in the initiation of the mammary gland of the embryo, duct formation, and alveolar development [62]. Hh signaling in mammals generally occurs between a signaling cell and a receiving cell. Mammals have three Hh ligands: Sonic hedgehog (Shh), Desert hedgehog (Dhh), and Indian hedgehog (Ihh) [63]. Hh signaling plays an important role in embryonic development, stem cell renewal, and repairing damaged mammary cellular tissue [64]. Hedgehog signaling plays a critical role in interactions between epithelium and stroma during ductal development as demonstrated by genetic analysis of two hedgehog signal transduction network genes, *Ptch1* and *Gli-2*. Defects in ductal morphogenesis occur following disruption of either gene. Many of the genes involved in hedgehog signaling are known oncogenes including *Smo*, *Shh, Gli-1*, and *Gli-2*. *Ptch1* can function as a tumor suppressor and has demonstrated the importance of hedgehog signaling in carcinogenesis. *Ptch1* depends on active hedgehog signaling and has been associated with several common cancers such as pancreatic, basal cell carcinoma, breast, ovarian, medulloblastoma, and small-cell lung carcinomas [65]. When MaSCs are grown as mammospheres several Hh family proteins are highly expressed including PTCH1, Gli1, and Gli2. During differentiation, these genes are down-regulated. Mammosphere-initiating cell numbers and mammosphere size are increased with increase in activation of Hh signaling and vice versa [66]. Normal development of mammary glands appears to be dependent on repression of the Hh pathway. As seen in mouse models, embryos that are null for either *Gli1* or *Gli2* have shown no apparent defects in mammary bud formation. On the other hand, constitutive activation of *Gli1* or lack of activated *Gli3* has resulted in mammary bud formation failure [66]. *Gli1* overexpression in mammary epithelial cells of the mouse resulted in a deficit in the function of the alveolar network, failure to lactate and, most significantly, in the emergence of hyperplastic lesions and the growth of tumors [67].

### p53

The tumor suppressor gene p53 has been discovered to play a role in stem cell maintenance. According to growing evidence, the loss of p53 has been linked to normal stem cell self-renewal, particularly following DNA damage. Work on mammary epithelial and hematopoietic stem cells provides an outstanding example of this. Unlike progenitor and differentiated cells, normal stem cells do not activate p53 in response to DNA damage. Interestingly, after DNA damage, p53-independent overexpression of p21 in normal stem cells limits p53 activity and switches cell divisions from asymmetric to symmetric self-renewal [68]. Given the key function of p53 in normal stem cell self-renewal and the hypothesis that cancer is a disease of excessive self-renewal, p53 mutations in CSCs could cause excessive self-renewal.The loss of p53 led to an increase in symmetric self-renewing divisions, which resulted in the proliferation of pre-malignant mammary stem cells, and when p53 was restored, the CSC pool was reduced due to the restoration of ACDs [69].

Another tumor-suppressive function of p53 is the control it exerts over the expansion of the number of SCs by regulating the cell division modality. Sherley and colleagues were the first to propose this in mammary epithelial cells, reporting a shift in in vitro growth kinetics from exponential to linear in an inducible nontumorigenic mouse cell line after p53 overexpression. This alteration in development pattern was consistent with asymmetric cell divisions and the creation of a quiescent stem cell pool [70]. Numb was identified as an upstream regulator of p53 in a later research which fueled speculation about p53's role in the regulation of ACD in mammary epithelial cells [71].

The most prevalent genetic variation found in human neoplasia is the p53 mutation. The p53 mutation is linked to a more aggressive form of breast cancer and a shorter overall survival time. However, the frequency of p53 mutations in breast cancer is lower than in other solid tumors. In breast tumors that express wild-type p53, changes in regulators of p53 activity and some downstream transcriptional targets of p53 have been found, which have been both genetic and epigenetic. A substantial proportion of people with the Li–Fraumeni cancer susceptibility syndrome, which increases the risk of breast cancer, have p53 mutations in their genes [72]. This suggests that p53 inactivation plays an essential role in mammary carcinogenesis, and the structure and expression of p53 in breast cancer have been extensively explored. Early research found that mutant p53 was expressed in breast cancer cell lines [73]. Loss of heterozygosity in the p53 gene has been found to be a prevalent occurrence in primary breast carcinomas [74].

### Musashi

Musashi-1 (MSI1) is essential for asymmetric cell division of sensory organ precursor cells [75] and is thought to have a key role in stem cell maintenance and differentiation [76]. MSI1 appears to serve as a translational repressor of target mRNAs encoding cell cycle inhibitory proteins, allowing stem cells to remain undifferentiated and self-renewing. MSI1 expression is also connected to stem cell over-proliferation in breast and intestinal malignancies [77].

MSI1 targets a number of genes involved in stem cell proliferation and cell cycle control. MSI1 expression has been shown to promote cancer cell proliferation in a variety of malignancies [78, 79]. MSI1 expression increases in CD133^+^ cancer stem cells in spheroid breast cancer cell cultures. By lowering MSI1 expression in spheroid culture, cancer stem cell proliferation is inhibited, as is the expression of Notch1 and cancer stem cell markers such as Oct4, Sox2, and c-Myc [80]. Breast cancer is a varied disease made up of various subtypes of breast cancer cells with varying morphological characteristics and clinical behaviors. Hormone receptor positive (ER/PR^+^) is a common subtype of breast cancer [81]. ER/PR^+^ cells can be self-renewing breast epithelial stem cells that divide asymmetrically to maintain self-renewal. In contrast to MSI1's activity in other cancers where it promotes cancer cell proliferation, MSI1 is responsible for epithelial-luminal transition in luminal tumors and luminal breast cancer cell lines. The shape of luminal breast cancer cells transforms to a basal-like appearance when MSI1 is downregulated by RNAi.

**Fig. 4:**
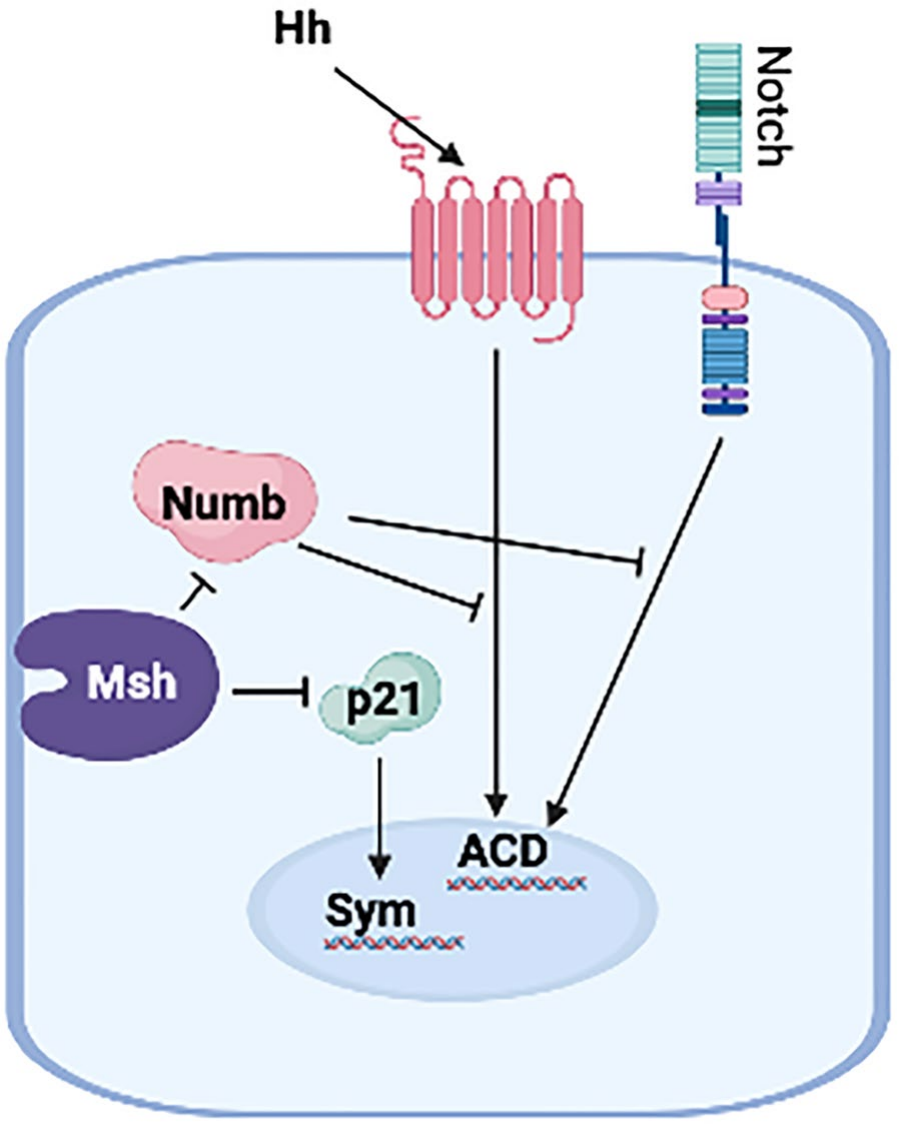
Effects on Notch, Numb, Musashi (Msh) and Hedgehog (Hh) pathways in controlling stemness. Numb inhibits both Hh and Notch regulated asymmetric cell division (ACD). Msh inhibits both Numb and p21 activity thereby inhibiting symmetric cell division and promoting ACD

Msi1 has been linked to translational regulation of the Numb gene and works as a positive Notch regulator (Fig. 4) [76]. Msi1 has been proposed as a possible MaSC compartment marker in the breast [82], and it has been demonstrated to stimulate luminal progenitor cell development in the normal mammary epithelial COMMAD cell line by activating the Notch and Wnt signaling pathways [83].

### Notch

Notch receptors are critical in controlling cell fate in a variety of tissues. Four homologous notch (Notch1-Notch4) proteins are present in mammals and are found expressed in a variety of stem and early-progenitor cells. Activation of the Notch pathway contributes to changes in cell fate, including stem cell self-renewal or segregation along a common lineage [84]. The involvement of the Notch pathway has been described during normal mammary gland development. Utilizing mammosphere mechanisms to investigate the role of Notch signaling in deciding mammary cell's future, findings indicate that Notch signaling is involved in several distinct mammary gland developmental stages. Notch serves as a regulator for decisions related to asymmetric fate of cells [51]. Notch1 and Notch2 have been found localized in the nuclei of prospective mammary progenitor cells [85]. Under the regulation of the mouse mammary tumor virus promoter, transgenic mice with a constitutively active Notch4 exhibited suspended mammalian gland growth and ultimately developed poorly differentiated adenocarcinoma [84]. The overexpression of Notch4's indigenous active role prevents the division of healthy in-vitro breast epithelial cells. The deregulation of normal mammary stem cell self-renewal may have involvement of abnormal Notch signaling, leading to carcinogenesis [51]. In vivo, transgenic mice which exhibit a constitutively active variant of Notch4 do not develop normal mammary glands and instead develop mammary tumors [86]. As Smith and colleagues have shown, in vivo, Notch4 plays a vital role in normal mammary growth and cancer formation [87]. In human breast epithelial stem cells Musashi-1 and Notch1 have recently been identified as two primary regulators of ACD (Fig. 4). Abnormal notch receptor expressions were demonstrated in different types of epithelial metaplastic defects and neoplastic defects, indicating that Notch may be functioning as a proto-oncogene. The vertebrate homologues Notch1 and Notch4 are involved in normal development of mammary glands and their mutated variations are associated with the development of mammary tumors in the mouse [84].

### Numb

The mammalian Numb gene produces at least four alternatively spliced transcripts, each of which produces protein isoforms ranging in size from 65 to 72 kDa [88]. These isoforms regulate cellular processes in different ways [89]. The presence of two sequence inserts within the PTB domain and the core region of the protein causes the distinct isoforms [90]. The four Numb isoforms are based on alternative splicing of two cassette exons: exon 3 and exon 9 [90]. Exon 9 is primarily included in stem cells and omitted in differentiated cells in the rat and human brain [88], mouse cerebral cortex [91], retina [92], and pituitary gland [93], where alternative splicing is developmentally regulated. Multiple cancer forms, including cervical squamous cell carcinoma [94], non-small cell lung cancer [95], urothelial carcinoma [96] and hepatocellular carcinoma [89] have increased expression of Numb exon 9 [89]. Activated MEK/ERK signaling enhances exon 9 inclusion in breast and lung cancer cell lines [97]. Activated MEK/ERK signaling enhances exon 9 inclusion in breast and lung cancer cell lines [97]. The mechanisms via which Numb's exon 9 contribute to cancer are unknown. Evidence suggests that the exon 9 may counteract the tumor-suppressive actions of Exon9sk Numb proteins, in addition to a potential gain-of-function acquired from increased Exon9in expression [98].

### Numb–Notch interactions

The NUMB–NOTCH relationship has been proposed as a major regulator of asymmetric cell division [9]. Notch transmembrane protein trafficking and endocytosis are controlled by Sanpodo, a membrane-associated protein that interacts with both NUMB and Notch [99, 100]. Numb/Notch signaling is essential in the balance between self-renewal and differentiation in *Drosophila* neural stem cells (NSCs). Because NOTCH and NUMB have evolved similar functions in cell fate determination and tumor angiogenesis, this biological pathway is a possible target for anticancer treatments. NUMB modulates oncogenic signaling pathways including p53, Hedgehog, and NOTCH (Fig. 4). Although the exact mechanism of NOTCH inhibition by NUMB is unknown, the intricacy of NUMB's isoforms and functions allows it to cover a wide range of roles in several signaling pathways.

### Wnts

Wnts are secreted, lipid-modified glycoproteins that activate multiple signal transduction pathways mediated by the cell surface receptors. Wnts control a broad range of cellular activities including determination of cell fate, proliferation, migration, polarity, and gene expression [101]. Wnt signaling induces plasticity for fate selection, extending the genetic mechanisms accessible to cells within the mammary genetic heritage. Wnt signaling is responsible for developing mammary fate in the embryonic ectoderm and also for the conservation of bi-potential basal stem cells in mature mammary ductal structures [102]. Experiments established that the dysfunction of the Wnt pathway directly leads to epithelial cancer in the epithelial stem cells of transgenic mice [103]. Overexpression of Wnt ligands in mammary stroma or active β-catenin in the epithelium of the mammary gland results in an increased number of mammary stem cells in transgenic mice [104]. Wnt1 signaling pathways are components of mammary stem cell self-renewal and involved in oncogenesis [105].

The seeming inconsistencies surrounding canonical Wnt signaling's role in stem cell self-renewal could be due to changes in Wnt levels or species-specific or cell type-specific variances [106–109]. These options are augmented by a number of tantalizing alternatives. One possibility is that Wnt/-catenin is involved in asymmetric cell divisions rather than self-renewal. Many progenitors divide asymmetrically to maintain a balance of dividing and differentiating cells within a tissue, giving one progenitor and one cell destined for differentiation [11, 110]. Wnt/-β-catenin-dependent asymmetric cell divisions discriminate and specify the fates of early progenitors in *Caenorhabditis elegans* embryos [111–113]. It was recently shown that if Wnt3a is administered to one side of a murine ES cell, β-catenin is distributed asymmetrically to its two daughter nuclei [114]. These findings imply that polarized canonical Wnt signaling may play a role in inducing asymmetric cell divisions, offering a possible explanation for how Wnts work in stem cells and differentiation. Furthermore, many stem and progenitor cells can divide symmetrically as well as asymmetrically [16, 115]. Since symmetrical divisions can occur when Wnt signaling is apolarized or too high in malignancies [116, 117], it's tempting to believe that when Wnt signaling is apolarized or too high, symmetrical divisions can occur, possibly causing both daughters to differentiate. If this is the case, changes in Wnt levels, combined with Wnts' ability to influence asymmetric cell divisions, could explain a slew of seemingly contradictory findings and shift attention away from species and cell type differences and toward differences in polarization and Wnt signaling levels perceived internally by the receiving stem cell/progenitor.

The Wnt receptor LRP5 is the first single biomarker to enrich for MaSCs and is also functionally implicated in stem cell maintenance [118]. Other putative transcriptional modulators or molecular pathways include Hedgehog, Bim-1, c-myc, and others, all of which impact MaSC activity in vitro or in vivo [119, 120]. The self-renewal and lineage commitment of MaSCs are governed by a complicated signaling channel network. Slug and Sox9 are governed by Notch signaling in a closed loop, while Bmi-1 functions directly downstream of Wnt-mediated c-myc or Hedgehog signaling, all of which contribute to MaSC self-renewal.

A Notch-Wnt synergy was recently described in a normal mammary environment, indicating a complicated interplay between Notch and Wnt in the interaction between mammary stem cells and the macrophageal niche [121]. Dll1 expression on mammary stem cells is critical for interactions with macrophages in the stromal environment [121]. Wnt ligands (Wnt10A, Wnt16, and Wnt3) are critical for MaSC numbers and activity, and macrophages express the ligands in response to Dll1 activation.

### Clinical implications of ACD in regards to cancer

Symmetric mitoses are required to replenish the stem cell pool in mature tissues. However, maintaining homeostasis requires rigorous control over the amount of cells with prolonged self-renewal capacity [122]. ACD regulatory disturbance of the key molecular players and pathways caused by genetic and/or functional changes may result in the growth of the stem cell pool and/or modification of the polarized architecture of a particular tissue, and hence may play a role in tumor initiation and development [123]. Abnormal tissue growth traits result from disrupted stem cell/progeny dynamics, which have been linked to cancer [123].

Disruption of apical determinants has similar effects in mammalian systems. By keeping Numb inactive and increasing Notch signaling, overexpression of PAR3 causes cells to divide symmetrically and retain stem-like features in both daughter cells [125]. Via transforming growth factor-beta (TGF-β) signaling, PAR6 has been identified as a causative factor for breast cancer epithelial–mesenchymal transition (EMT). In mice, mutated PAR6 inhibits TGF-β signaling and prevents mammary tumor lung metastasis [126].

Wnt is a confirmed proto-oncogene that has been related to breast cancer, medulloblastoma, and other types of neoplasia. Wnt dysregulation enhances β-catenin, nuclear localization, which leads to target gene expression, that promotes tumor growth and is linked to a poor prognosis in several malignancies. Wnt signaling is also a major driver of EMT [114], which may be caused in part by asymmetric cell division dysregulation. Early tumorigenic lesions of MMTV-Wnt1 transgenic mice are maintained by WNT signaling [127]. Furthermore, the extracellular matrix component periostin enhances the maintenance of cancer stem cells by recruiting WNT [128]. Inhibiting Hedgehog signaling affects the ability of mammary CSCs to generate primary spheres as well as their ability to self-renew and form secondary spheres in the mammary system [119].

There is evidence to support the role of ACD dysregulation in breast cancer from a molecular standpoint, but a direct demonstration of a causal relationship between the two has been difficult [27]. Disruption of signaling networks involved in asymmetric division leads to a proliferative state and an accumulation of stem-like cells with limited differentiation potential in neoplastic disease. For example, a constitutively active mutant version of Drosophila aPKC promotes Notch signaling by reducing active Numb on both the apical and basal sides, boosting neuroblast self-renewal and tumor formation [32, 124].

ACD has been observed in multiple forms of cancers including glioblastoma and colon cancers [139–142]. In a pre-clinical model of glioblastoma multiform (GBM), ACD results in the generation of daughter cells with EGFR-mediated enhanced therapeutic resistance [139]. CD133 and Numb also regulate ACD by glioma cells [140]. In preclinical models of colon cancer Numb and Notch are demonstrated regulators of ACD by the cancer cells [141, 142]. These results indicate that ACD in cancer cells is regulated by similar mechanisms of ACD found in normal stem cells.

#### Techniques used to study stem/progenitor cells in mammary glands

ACD relies on the differential portioning of niche contacts and fate determinants. Tracking the division orientation and determinant partitioning is a strong indicator of asymmetry if the nature of the niche and determinants for the system under investigation are clearly established. Live imaging offers the best perspective into its workings by observing the movement of determinants and the location of the daughter cells in space and time after cytokinesis [129].

Permanent and inheritable genetic markers can be used to label the cells that express a SC-specific promoter such as GFP, RFP, YFP, mCherry genes. The distribution of markers within a tissue or a whole organism is tracked for a long term. When all distinct lineages can be traced back to a single cell, that cell is considered as a multipotent stem cell [130]. While there are restrictions to direct examination of the lineage in whole organisms and tissues, these do not extend to lineage study of single cells in culture.

The premise that a spheroid composed of cells at various differential stages can only be generated by cells with SC properties allows researchers to expand and study a stem cell population in vitro. Such spheres are called mammosphères in the case of MaSCs. Mammospheres can be sequentially passaged and sphere-forming efficiency (SFE) is demonstrated as a measure of their capacity to self-renew. SCs which segregate primarily in an asymmetric manner will decrease their SFE slowly before full culture saturation occurs, while SCs dividing predominantly symmetrically will grow indefinitely. Using the PKH26-based label retention assay, SCs and progenitors within mammospheres can be isolated to near homogeneity. PKH26, a lipophilic dye, marks the cell membrane and separates it from the cell divisions. In MaSCs (~ 1:4), quiescent or slowly dividing cells are fortified and retain the marker during the assay. PKH26 retention has been used to evaluate the SC division mode, because ACDs produce one cell which stays silent and therefore maintains the original identity, and another which continues to divide. The dye would also be diluted rapidly in the culture of MaSCs where stem cell divisions are prevalent. Additionally, the PKH-negative fraction of cells will activate a mammary culture and reform a mammary gland after transplantation [38, 69, 131]. Therefore, mammospheres can be passed in sequence, and the output of the sphere formation is expressed as an indicator of their self-renewal ability [27].

Primary stem cells isolated from various tissues can be expanded as clones with design details identical to the original organ. The trend of segregation that promotes organoid morphogenesis can be credited to self-renewing ACD [132]. The stem cells can be coaxed into forming structures that contain clusters of cells when positioned inside a hydrogel (often Matrigel) and in the presence of suitable exogenous factors. The development of organoid stem cell systems to produce 3D self-organized tissue models offers a convincing new class of biological model to act as tissue and organ proxies [133]. Organoids capture a broad variety of biological parameters including the spatial arrangement of heterogeneous tissue-specific cells, cell–cell communications, cell–matrix interactions, as well as other tissue-specific cell physiological functions within the organoid. Organoids fill gaps in current model systems by offering a robust mechanism capable of prolonged cultivation and manipulation, thus making in vitro physiology more reflective of in vivo [134].

Organ reconstitution upon transplantation is considered the gold standard of stem cell experiments. The capacity of a recipient host to reconstitute a tissue has been used extensively to determine the frequency of SCs in a population group. The development of a completely differentiated mammary outgrowth is assessed 10–14 weeks after cells are transplanted into the prepubertal gland devoid of endogenous epithelium. The transplantation process uses a serially diluted quantity of cells to be delivered into the unobstructed fat pad of prepubertal mice to reduce the dilution conditions. This makes the calculation of the size of the stem cell pool in a population group and the determination of whether a single stem cell may be sufficient to reconstruct the gland tissue.

### Direct imaging

Stem cells self-renew by dividing asymmetrically. Stem cells retain their template DNA strands during asymmetric division, while the newly synthesized DNA strands separate into newly created daughter cells by selectively isolating the template DNA strands from the mutations associated with DNA replication and the resulting risk of cancer [85].

A corollary to this theory holds that incorporated analog thymidine labels are retained by somatic stem cells due to slow division rate or asymmetric DNA segregation [135]. This closely regulated method was initially used with radiolabeled nucleoside analogs and eventually observed utilizing light microscopy with analogues that enabled newborn cell monitoring and their subsequent characterization. In the strictly regulated S-phase of the cell cycle, cells which replicate undergo DNA synthesis. Analogs of pyrimidines such as deoxynucleotide thymidine may be incorporated into DNA replication, effectively labeling the dividing cells to allow their characterization. Past research has shown that self-renewing mammary stem cells arise during puberty-associated allometric growth in mammary ducts. In the study conducted by Park et al. during allometric ductal expansion, they conducted a 2-week long labeling period that labeled newly forming mammary stem cells [85]. Cells which integrated and maintained the nuclear label are label retaining cells (LRCs) after prolonged chase time frames. A second nuclear marker, 5-bromodeoxyuridine, was administered before euthanasia to identify cells that were progressing through the cell cycle. Sorting of the mammary cells obtained after euthanasia was based on nuclear mark preservation. Mammary LRCs differently expressed members of the Notch and Wnt signaling pathways. Both pathways are involved in the regulation of stem cells in the mammary gland of the mouse. In the mouse mammary gland, LRCs have been reported in duct development during expansion of puberty, in alveolar structures that form during pregnancy, and in surrounding mammary stroma and blood vessels [6, 7, 85]. The mammary LRCs are presumed to be mammary stem and progenitor cells [6, 7, 85]. Booth et al. showed that there is a subpopulation of LRC in the mouse mammary gland that persists during alveologenesis [7]. During pregnancy, these cells respond to hormonal signals and join the cell cycle while selectively maintaining their original template DNA. Additionally, periductal or peri-acinar positions are found in nonepithelial LRCs. During pregnancy, these LRCs enter the cell cycle. Newly formed label-retaining epithelial cells emerge inside the growing alveoli during alveologenesis and continue to cycle and maintain their original DNA template strands as determined by a classic pulse-chase experiment [7]. Thymidine analog labeling is based on the premise that immortal DNA templates are marked during symmetric stem cell divisions and that successive divisions, in which the marked template is maintained, are asymmetric. Nonetheless, during development and throughout adulthood, stem cells may undergo symmetric, asymmetric, or a mixture of symmetric and asymmetric divisions. Therefore, timing of label delivery is important for proper experiment execution. The thymidine analog labeling must include accurate time knowledge all through expansion or regrowth when stem cells alter from symmetric divisions to asymmetric ones. Mistimed administration or removal of a label will result in its failure to incorporate DNA strands or premature dilution into the marked templates to differentiating daughter cells [136].

### Indirect labeling

*Bioluminescence imaging* (BLI), is an optical imaging technique of indirect cell labeling with reporter genes. This is a promising approach for monitoring cells in small animal models. Bioluminescence is produced by the action of luciferase enzymes and their substrates in live organisms, when chemical energy is converted into visible light. BLI has been a vital tool for the empirical assessment of the fate of stem cells that have been transplanted by marking of the cells with a constitutive bioluminescent reporter gene. Nonetheless, owing to lower tissue infiltration of light photons and insufficient quantification due to in vivo tissue absorption and dispersion in living tissue, restricting its use in animals larger than rats [137]. BLI technologies which provide more sensitive, quantitative and three-dimensional information are also under development [138].

## Conclusion

Asymmetric stem cell division is a widespread process in various living organisms, and it is critical for the fate of cell lineages. Its balance with symmetrical cell division is vital for long term tissue homeostasis, failure of which may lead to carcinogenesis. In mammary stem cells, molecular mechanisms important for ASD are cell polarity, fate determinants, and orientation of mitotic spindle.

## Data Availability

Not applicable.

## Abbreviations

^3^HTdR: Tritiated thymidine
5BrdU: 5-Bromo-deoxyuridine
ACD: Asymmetric cell division
BLI: Bioluminescence imaging
aPKC: Atypical protein kinase C
CNS: Central nervous system
CSC: Cancer stem cell
Dhh: Desert hedgehog
DNA: Deoxyribonucleic acid
EMT: Epithelial–mesenchymal transition
ER: Estrogen receptor
GFP: Green fluorescent protein
Hh: Hedgehog
Ihh: Indian hedgehog
LRC: Label retaining cell
LREC: Label retaining epithelial cell
MaSC: Mammary stem cell
MMTV: Mouse mammary tumor virus
MSI1: Musashi-1
NB: Neuroblast
PR: Progesterone receptor
RFP: Red fluorescent protein
SC: Stem cell
SFE: Sphere forming efficiency
Shh: Sonic hedgehog
TEB: Terminal end bud
TGF: Transforming growth factor
YFP: Yellow fluorescent protein
